# The Glucocorticoid Receptor Is a Critical Regulator of HIV Latency in Human Microglial Cells

**DOI:** 10.1007/s11481-018-9798-1

**Published:** 2018-07-10

**Authors:** David Alvarez-Carbonell, Fengchun Ye, Nirmala Ramanath, Curtis Dobrowolski, Jonathan Karn

**Affiliations:** Department of Molecular Biology and Microbiology, Case Western Reserve University, Cleveland, OH 44106 USA

**Keywords:** Microglial activation, Glucocorticoids, Glucocorticoid receptor, HIV-induced neurocognitive disorders, HIV-associated neurocognitive disorders, Dexamethasone, TNF-α

## Abstract

We have developed models of HIV latency using microglia derived from adult human patient brain cortex and transformed with the SV40 T large and hTERT antigens. Latent clones infected by HIV reporter viruses display high levels of spontaneous HIV reactivation in culture. BrainPhys, a medium highly representative of the CNS extracellular environment, containing low glucose and 1% FBS, reduced, but did not prevent, HIV reactivation. We hypothesized that spontaneous HIV reactivation in culture was due to the expression of pro-inflammatory genes, such as TNF-α, taking place in the absence of the natural inhibitory signals from astrocytes and neurons. Indeed, expression and secretion of TNF-α is strongly reduced in HIV-latently infected microglia compared to the subset of cells that have undergone spontaneous HIV reactivation. Whereas inhibitors of NF-κB or of macrophage activation only had a short-term silencing effect, addition of dexamethasone (DEXA), a glucocorticoid receptor (GR) agonist and mediator of anti-inflammation, silenced the HIV provirus in a long-term, and shRNA-mediated knock-down of GR activated HIV. DEXA also decreased secretion of a number of cytokines, including TNF-α. Chromatin immunoprecipitation analysis revealed that DEXA strongly increased GR occupancy at the HIV promoter, and reduced histone 3 acetylated levels. Moreover, TNF-α expression inhibitors in combination with DEXA induced further HIV silencing and increased the histone 3 lysine 27 tri-methylated epigenetic mark of repression at the HIV promoter region. We conclude that GR is a critical repressor of HIV transcription in microglia, and a novel potential pharmacological target to restrict HIV expression in the CNS.

**Electronic supplementary material:**

The online version of this article (10.1007/s11481-018-9798-1) contains supplementary material, which is available to authorized users.

## Introduction

The CNS remains a sanctuary for ongoing HIV replication, even when full viral suppression has been achieved in the peripheral blood using potent anti-retroviral therapy (ART) (Ellero et al. 2017). HIV-associated neurocognitive disorders (HAND) are a compendium of dysfunctions associated with HIV infection in the brain. HAND, which affects about one third of HIV patients (Tozzi et al. 2007; Heaton et al. 2010), can be considered to be a milder form of HIV-associated dementia (HAD) (Farhadian et al. 2017), and is a major unresolved clinical manifestation in well suppressed patients that urgently needs to be ameliorated.

HIV infection in the CNS disrupts normal neuronal function through the production of neurotoxic viral and host factors, as well as microgliosis (Walsh et al. 2014). The two most important neurotoxic viral factors are the viral envelope, gp120 (Toggas et al. 1994; Tenneti and Lipton 2000; Iskander et al. 2004; Kaul and Lipton 2005), and the viral transactivator, Tat (Nath 2002; Bruce-Keller et al. 2003; Chauhan et al. 2003). Neurotoxic host factors that, together with HIV proteins, exacerbate neuronal damage include cytokines and chemokines produced by infiltrating macrophages, astrocytes and activated microglia (Ellis et al. 2009). Specifically, activated microglia release the pro-inflammatory cytokines interleukin (IL)-1β, IL-6, interferon (IFN)-γ, and tumor necrosis factor-α (TNF-α) (Sawada et al. 1989; Mizuno et al. 1994; Suzumura et al. 1996; Frucht et al. 2001; Mizuno et al. 2003), which promote neuronal damage. We have demonstrated that IL-1β and TNF-α are strong stimulators of HIV transcription in microglia (Alvarez-Carbonell et al. 2017; Garcia-Mesa et al. 2017). HIV reactivation in microglial cells is likely to be further enhanced by autocrine TNF-α loop signaling inducing constitutive NF-κB activation (Pekalski et al. 2013).

In normal circumstances, microglial activation in response to CNS infections is protective. For example, activated microglia can attenuate excitotoxic of ischemic injury in rodents (Berezovskaya et al. 1995), prevent neuronal apoptosis in cell culture (Toku et al. 1998), induce neurite growth and after-injury recovery (Lazarov-Spiegler et al. 1996; Prewitt et al. 1997; Rabchevsky and Streit 1997; Batchelor et al. 1999), and protect neurons through the release of neuroprotective factors such as fibroblast growth factor (Araujo and Cotman 1992), nerve growth factor (Heumann et al. 1987) or IL-4 (Butovsky et al. 2005), or by activation of specific targets such the glucocorticoid receptor (NR3C1 or GR) (Heikinheimo et al. 1987; Blind and Garabedian 2008). These protective microglial responses are strictly limited by intrinsic molecular mechanisms that limit neurotoxicity (Boillee et al. 2006; Streit 2006; Neumann and Takahashi 2007).

Unfortunately, a chronic pro-inflammatory response leads directly to neurodegeneration due to excessive secretion of cytokines and chemokines (Streit 2006). Therefore, microglia-mediated neurotoxicity, as seen in diseases such as HAND, is likely to be the result of excessive and uncontrolled stimulation (van van Rossum and Hanisch 2004; Cardona et al. 2006), and/or impaired intrinsic molecular mechanisms limiting microglial activity (Boillee et al. 2006; Streit 2006; Neumann and Takahashi 2007).

We have recently described a method for establishing immortalized human microglial cells (hμglia) (Garcia-Mesa et al. 2017). These cells have microglia-like morphology, express key microglial surface markers, display appropriate migratory and phagocytic activity, and are able to mount an inflammatory response characteristic of primary microglia. We used hμglia to generate stable cell lines latently infected with HIV proviruses (hμglia/HIV) (Alvarez-Carbonell et al. 2017; Garcia-Mesa et al. 2017), and showed that they can respond to specific inflammatory activation signals, including the toll-like receptor (TLR) agonists (Alvarez-Carbonell et al. 2017). These novel and extensively characterized cell lines are proving to be important tools to study microglial cell function and molecular mechanisms involved in HIV transcription in CNS.

In contrast to the T-cell models of HIV latency that we have also developed (Friedman et al. 2011; Jadlowsky et al. 2014; Nguyen et al. 2017), latently infected microglial cells show progressive HIV reactivation in culture and, therefore, are represented by an authentic latent population (GFP^−^ cells), where the viral DNA is completed silenced, and a fully activated population (GFP^+^ cells), where HIV is expressed. We hypothesized that, under ex vivo culture conditions, the microglial cells are not receiving adequate inhibitory signals to maintain quiescence. Therefore, over time, more and more cells start producing pro-inflammatory molecules, including TNF-α, resulting in HIV reactivation. Here, we identify specific cell culture conditions, which more closely resemble CNS physiological environment, that can significantly decrease basal level of HIV expression, and permit long-term HIV silencing.

The interaction between glucocorticoids (GC) and GR in microglia plays a major role in protecting the brain against innate immune response (Nadeau and Rivest 2003). This control mechanism seems to be compromised during many neuropathological conditions, including Parkinson disease (PD) (Heikinheimo et al. 1987; Morale et al. 2004; Sugama et al. 2009) and brain ageing (Nichols et al. 2001; Murphy et al. 2002). Once bound to GC, GR/GC regulates gene expression by binding to hormone-response elements in the promoter of various genes alone or in combination with other transcription factors such as NF-κB or AP-1 (Lazar Jr. et al. 1992). Here, we show that activation of the GR by dexamethasone (DEXA), a synthetic glucocorticoid, which is also able to impair the ability of rat dendritic cells to produce IL-1β and TNF-α (Avenant et al. 2010), led to potent repression of HIV transcription. Dysfunctional regulation of HIV by GR may therefore contribute to HIV replication in the CNS, and the eventual development of HAND.

## Materials and Methods

### Microglia Culture Medium

Unless otherwise noted, hμglia/HIV cells were cultured in BrainPhysTM medium (StemCell Technologies, Canada) containing 1X N2 supplement-A (Gibco-Invitrogen, #17502–048), 1X penicillin streptomycin (Gibco ™, #15140122), 100 μg/mL normocin™ (Invivogen, #ant-nr-1), 25 mM glutamine (Gibco ™, #25030081), 1% FBS, and 1 μM DEXA (freshly added to the cell culture) (Sigma-Aldrich, # D4902).

### Chemicals & Reagents

TNF-α releasing inhibitors nedocromil sodium (NaN) and sodium cromoglycate (NaC), TNF-α production antagonist thalidomide (TLM), and synthetic GC DEXA and mifepristone, were purchased from Sigma-Aldrich (St. Louis, MO, USA). To manipulate HIV expression, which was measured by flow cytometry of GFP, in hμglia/HIV cells, TNF-α (Invitrogen, #PHC3015), IL-1β (Sigma, #I9401), poly (I:C) (Invivogen, #tlrl-pic), LPS (Invivogen, #tlrl-eklps), (d) DHMEQ (MedChem Express, #HY-14645A), (s) DHMEQ (MedChem Express, #HY-14645), IKKγ NEMO binding domain inhibitory peptide (Imgenex, CA), TGF-β1 (Sigma-Aldrich), and anandamide (Sigma-Aldrich, #A0580) were used. Rabbit monoclonal antibodies to glucocorticoid receptor (GR), phospho-glucocorticoid receptor (Ser226) (P-GR) and RNA polymerase II (RNA pol II), and rabbit polyclonal antibodies to acetylated histone 3 (H3Ac) and tri-methylated histone 3 (H3K27me3) were purchased from Cell Signaling Technology (Danvers, MA, USA). Rabbit polyclonal antibodies to HIV Tat and tubulin proteins were purchased from Abcam (Cambridge, MA, USA).

### Expression of TNF-α and Other Cytokines

To measure TNF-α expression, GFP^+^ and GFP^−^ cells from hμglia/HIV HC69 microglia, present at an approximate ratio of 6 GFP^−^ to 4 GFP^+^, were separated by sorting and propagated in culture. Identical number (2 × 10^5^) of the resulting GFP^+^ and GFP^−^ cells were seeded in 12-well plate. Upon stimulation with various doses of poly (I:C) (1, 10, 100, 500, and 1000 pg/mL) for 24 h (h), the supernatant from each well was collected and centrifuged at 10,000 g for 5 min to remove cellular debris. The concentration of TNF-α in the supernatants was measured by using a human TNF-α ELISA kit from Sigma-Aldrich and following instruction of the manufacturer. Each measurement was conducted in triplicate. Meanwhile, total RNA from cells of each well was isolated and purified by using an RNA purification kit from Qiagen (Hilden, Germany), and converted into cDNAs by using a reverse transcription kit from Bio-Rad (Hercules, CA, USA). The relative level of TNF-α mRNA in each sample was measured by quantitative reverse transcription-polymerase chain reaction (qRT-PCR), using primers 5’-ATGAGCACTGAAAGCATGATCC-3′ (forward) and 5’-GAGGGCTGATTAGAGAGAGGTC-3′ (reverse). The mRNA level of the housekeeping gene β-actin in each sample was used as reference for normalization, which was measured by qRT-PCR using the primers 5’-TCCTCTCCCAAGTCCACACAGG-3′ (forward) and 5’-GGGCACGAAGGCTCATCATTC-3′ (reverse). Each qRT-PCR was conducted in triplicate in triplicated samples. To investigate the effect of DEXA on TNF-α expression, TNF-α secretion and mRNA were measured in unsorted HC69 cells either untreated or treated with DEXA (1 μM) for 24 or 72 h, using TLM (25 μM) as positive control. In addition, secretion of other cytokines was measured by using the Human Cytokine Array Kit, Panel A (R&D Systems, #ARY005), and following the manufacturer instructions, after sorting GFP^+^ and GFP^−^ cells, as above, and exposing or not each population to 1 μM DEXA for 96 h.

### shRNA-Mediated Knockdown of GR

Lentiviral particles expressing scrambled control shRNA or GR [RHS4533-NM_00113509] specific shRNA (Vector Builder, CA) were used to infect 1 X 10^5^ HC69 cells, respectively. Three days after infection, drug-resistant cells were selected in medium containing blasticidin (2 μg/mL). GFP expression of the resulting cells was analyzed by flow cytometry and knocking down of GR was verified by Western blot analysis.

### Western Blot Analysis

For determining the total expression of GR and Tat in shRNA-treated cells as well as of GR, P-GR, and Tat in cells un-exposed or exposed to DEXA, whole cell extracts (WCE) were prepared from 1 X 10^5^ cells in RIPA buffer (25 mM Tris, pH 7–8, 150 mM Na, 0.1% SDS, 0.5% sodium deoxycholate, 1% Triton X-100). Western blot analysis was carried out using the ECL system. As loading control, we measured the expression of tubulin.

### Chromatin Immunoprecipitation (ChIP) Analysis

Sample preparation for ChIP experiments was carried out essentially as previously described (Alvarez-Carbonell et al. 2017), with some modifications. For each experimental condition, 7 × 10^6^ HC69 cells were plated on a 150-mm diameter plate and incubated overnight. In one experiment, unsorted cells were untreated or treated with DEXA (1 μM) for 16 h (short-term). In a second experiment, GFP^+^-sorted cells were left untreated or treated with either NaN, NaC or TLM (25 μM) either in the absence or presence of DEXA (2.5 μM) for 96 h (long-term). The cells were then cross-linked in 1% formaldehyde, incubated for 10 min at ambient temperature, and the reaction quenched by adding glycine 1 mM, and further incubated for 5 min. Chromatin for IP was then prepared, and fragmented chromatin exposed to 5 μg of control IgG or anti-RNAP II, anti-GR, anti-H3-Ac or anti-H3K27 antibodies. 45 μL of the chromatin fractions were then diluted in 100 μL of IP dilution buffer, and added to antibody-coated well. Antibody binding reactions were carried out for 1 h at ambient temperature with 500 rpm shaking. After two washes with RIPA buffer and one wash with TE buffer, chromatin-IgG complexes were eluted and digested in elution/Proteinase K buffer for 30 min at 65 °C. Freed DNA was purified with PCR magnetic clean up beads (PCR cleanup beads, Axygen). Following purification, the ChIP DNA was subjected to qPCR using primers for the HIV promoter region (−116 forward /+4 reverse) as described previously (Friedman et al. 2011). As a positive control for GR recruitment, we examined GR binding to the promoter of the human dual specificity protein phosphatase 1 (DUSP1), also known as mitogen-activated protein kinase phosphatase-1 (MKP-1) and a well-established GR responsive gene (Reddy et al. 2009; Shipp et al. 2010). The GR binding region on the DUSP1 gene promoter was measured using the primers forward (−1428) 5’-CAGAAGTTGCCACTGGTGAT-3′ and reverse (−1118) 5’-CGTTATAGGCCGAAAGCAAA-3′. As negative control, a GR non-binding region on the same gene was measured with the primers forward (−106) 5’-CCGTCACGTGATCACCATT-3′ and reverse (−16) 5’-GCGTTTATATGCGGCCTCT-3′. All qPCR reactions were conducted in triplicate in triplicated samples.

### HIV Expression in Multiple μglia/HIV Clones

Inhibition of HIV expression was measured by flow cytometry in a variety of clonal populations derived from μglia/HIV cells after sorting for GFP^+^ cells, and culturing them for 72 h with the following treatments: control, 25 μM NaN, 25 μM NaC and 25 μM TLM in either the absence or presence of 2.5 μM DEXA.

## Results

### Spontaneous Reactivation of Latently Infected Microglial Cells in Culture

**Fig. 1:**
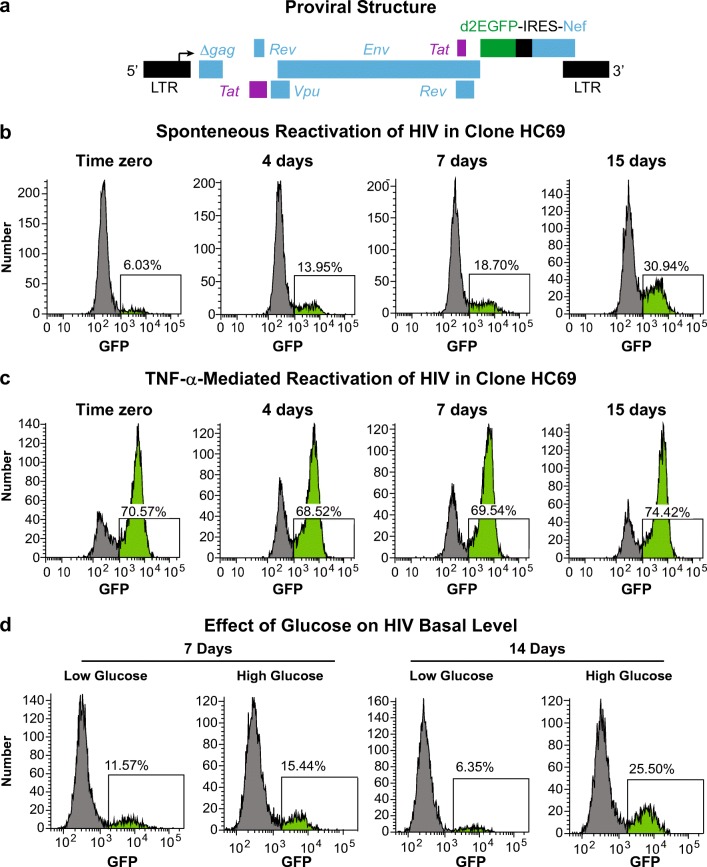
Spontaneous HIV emergence from latency in infected human microglia. (**a**) Genomic organization of the HIV lentiviral vector. A fragment of HIV-1_pNL4–3_, containing *Tat*, *Rev*, *Env*, *Vpu*, and *Nef* with the reporting gene d2EGFP, is cloned into the pHR’ backbone. The resulting plasmid was used to produce the VSVG HIV particles, as described previously (Kim et al. 2006). **b** Flow cytometry analysis of HIV expression in the representative clone HC69 (Alvarez-Carbonell et al. 2017; Garcia-Mesa et al. 2017; Llewellyn et al. 2017) at Time zero, 4, 7 and 15 days. **c** Reactivation of HIV at the indicated time points with TNF-α at 100 pg/mL. **d** Flow cytometry analysis of HIV expression in HC69 cells exposed to low (1 g/L) or high (4.5 g/L) glucose concentration for either 7 or 14 days. GFP^+^ cell populations are indicated in *bright green*, and the proportion of GFP-expressing cells is indicated in *percentage*

We have derived multiple clonal cell lines of hμglia latently infected by HIV. The hμglial cells were infected with vesicular stomatitis virus G-(VSVG) pseudotyped lentiviral vectors expressing Tat, Rev., Env, and Vpu, and carrying a short-lived green fluorescence protein (d2EGFP) upstream of Nef (Fig. 1a). This allows monitoring of viral transcription by fluorescence-activated flow cytometry (FACS) and/or fluorescence microscopy (Wires et al. 2012; Alvarez-Carbonell et al. 2017; Garcia-Mesa et al. 2017; Llewellyn et al. 2017).

HIV expression in a representative clone, HC69 (Alvarez-Carbonell et al. 2017), grown in DMEM/high glucose (Alvarez-Carbonell et al. 2017; Garcia-Mesa et al. 2017; Llewellyn et al. 2017), was monitored by flow cytometry for 2 weeks as shown in Fig. 1b. At the start of the culture (Time zero), only ~6% of the cells expressed HIV, however, in contrast to latently infected Jurkat T-cell clones (Jordan et al. 2003; Weinberger et al. 2005; Pearson et al. 2008; Singh and Weinberger 2009), where spontaneous activation levels reach an equilibrium in the population, HIV expression in the microglial cells increased progressively reaching ~31% after 15 days. Treatment of these cells at each of these time points with 100 pg/mL of TNF-α for 16 h induced HIV expression up to a maximum of ~ 70% at each time point (Fig. 1c), illustrating that there was no loss of HIV proviruses or their transcriptional capacity under these culture conditions.

### Optimal Medium to Restrict Spontaneous HIV Reactivation

The unusual progressive reactivation seen the microglial cell system suggested to us autocrine mechanisms might be responsible. Accordingly, we sought culture conditions that would minimize the spontaneous exit of HIV from latency. We originally cultured microglial cells in DMEM with a glucose concentration of 4.5 g/L or 25 mM, which is much higher than circulating glucose levels. (Silver and Erecinska 1994; Abi-Saab et al. 2002; de Vries et al. 2003; Bardy et al. 2015). Therefore, we measured the effect of reduced glucose level in the culture medium on HIV expression. We found that in DMEM/low glucose (1 g/L or 5.55 mM), basal level of HIV expression is maintained at ~11% after 7 days, and significantly decreased down to ~6% after 14 days, whereas in high glucose (4.5 g/L), HIV expression is ~15% after 7 days and increased up to ~26% after 14 days (Fig. 1d). Glucose concentrations below 0.5 g/L resulted in excessive loss of cell viability (supplement Fig. 1). We also measured the effect of adding HEPES to the media, to maintain right pH control, on basal level of HIV expression in HC69 cells. After 7 days, the presence of this buffer induced HIV expression from ~7% up to ~14%, and after 14 days HIV level was ~17% (supplement Fig. 2). Also, reducing fetal bovine serum (FBS) to 1% preserved cell viability and helped to minimize spontaneous HIV reactivation (supplement Fig. 3). In all these experiments and the following experiments, the medium was changed every 2 to 3 days.

A new medium formulation, BrainPhys (Bardy et al. 2015), has been developed to support in vitro neuronal activity by more closely mimicking the in vivo brain physiological conditions. We tested the above conditions in the BrainPhys medium background. Our results (Table 1) showed that in the BrainPhys-based medium HIV latency is well preserved. After 21 days, a 5% basal expression was observed, which is slightly better than in DMEM-based medium containing low glucose level (1 g/L). This may be because BrainPhys medium has a glucose content of only 2.5 mM (~ 0.5 g/L) (Bardy et al. 2015).

**Table 1:** Hμglia/HIV HC69 cells growth media

Base Medium	N2 Supplement	Pen Strep	Normocin	Glutamine	FBS	DEXA (freshly added)	HIV Expression After 1 week	HIV Expression After 2 weeks	HIV Expression After 3 weeks
DMEM high glucose	1X	1X	100 μg/mL	–	1%	1 μM	19% ± 2.5%	16% ± 4.5%	15% ± 2.8%
DMEM low glucose	1X	1X	100 μg/mL	–	1%	1 μM	15% ± 1.8%	10% ± 3.5	8% ± 2.1%
BrainPhys	1X	1X	100 μg/mL	2.5 mM	1%	1 μM	13% ± 2.6%	8% ± 1.4%	5% ± 3.3%

### HIV Reactivation Correlates with TNF-α Expression in Microglial Cells

**Fig. 2:**
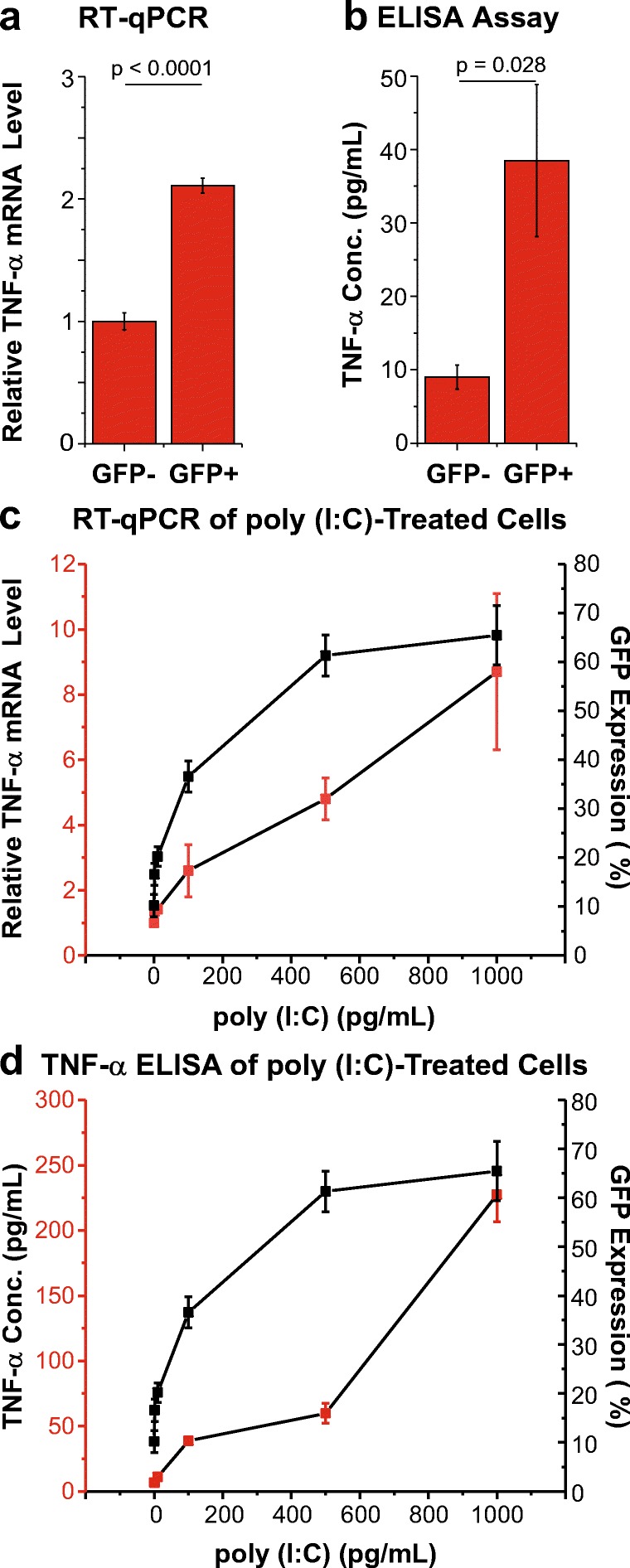
*Reactivation of latent HIV in microglial cells is featured with elevated production of TNF-α.****a****Relative levels of TNF-α mRNA (Y-axis) in GFP*^*−*^*and GFP*^*+*^*cells (X-axis) separated by sorting, which were measured by qRT-PCR.****b****Levels of secreted TNF-α protein (Y-axis) in the culture supernatants of equal numbers of GFP*^*−*^*and GFP*^*+*^*HC69 cells at 4 days after sorting were measured by ELISA.****c****Relative TNF-α mRNA levels (Y-axis) and****d****secreted TNF-α concentration (Y-axis) determined from GFP*^*−*^*cells that were exposed to increasing doses of poly (I:C), as indicated (X-axis), for 16 h. In each case, the e*rror bar represents the standard deviation of the sample (Excel) of three different experiments

Since our previous studies showed that microglial cells can produce TNF-α, and that TNF-α is a potent inducer of latent HIV through the NF-κB pathway (Alvarez-Carbonell et al. 2017; Garcia-Mesa et al. 2017), we tested the hypothesis that the GFP^+^ cells may become activated due to autocrine activation by this cytokine. In order to compare activated and inactivated cells, we sorted a spontaneously reactivated cell population into GFP^−^ and GFP^+^ fractions. As shown in Fig. 2a, b, the GFP^+^ cells contained ~2 times the levels of TNF-α mRNA and secreted ~4 times more TNF-α than the GFP^−^ cells. Treatment of the GFP^−^ cells by poly (I:C) stimulated TNF-α mRNA production and TNF-α secretion (Fig. 2c, d), although this occurred at higher concentrations than the direct activation of the HIV provirus as measured by GFP expression, consistent with our previous results showing that IRF-3 induced by poly (I:C) can directly activate latent HIV proviruses (Alvarez-Carbonell et al. 2017).

### GR Activation Decreases Basal Level of HIV Expression

In order to identify inhibitors that could suppress cytokine production by the microglial cells and thereby help to maintain low basal level of HIV expression under normal cellular growth, we screened various compounds that have been reported to suppress macrophage activity:

1. To evaluate the impact of NF-κB, we tested the inhibitors (d) dehydroxymethylepoxyquinomicin (DHMEQ) and (s) DHMEQ, the eutomer of DHMEQ, which inhibits NF-κB activation with an IC50 value lower than (d) DHMEQ (Matsumoto et al. 2000), and the IKKγ NEMO binding domain inhibitory peptide (Wires et al. 2012).
2. TGF-β1 inhibits the proliferation of microglia and prevents their production of reactive oxygen (e.g. superoxide (O^2−^)) and nitrogen (e.g. nitric oxide (NO)) intermediates (Wahl et al. 2006). We therefore examined anandamide (*N*-arachidonoylethanolamine (AEA)), an immune modulator in CNS, that attenuates LPS-induced NO release in microglia (Malek et al. 2015).
3. To evaluate the impact of GC, we tested the synthetic GC DEXA, which represses the expression of inflammatory gene products including IL-1β and TNF-α (Newton 2000), and mifepristone, a GR antagonist (Heikinheimo et al. 1987).

The effect of these compounds on the basal level of HIV expression was monitored for 45 days.

**Fig. 3:**
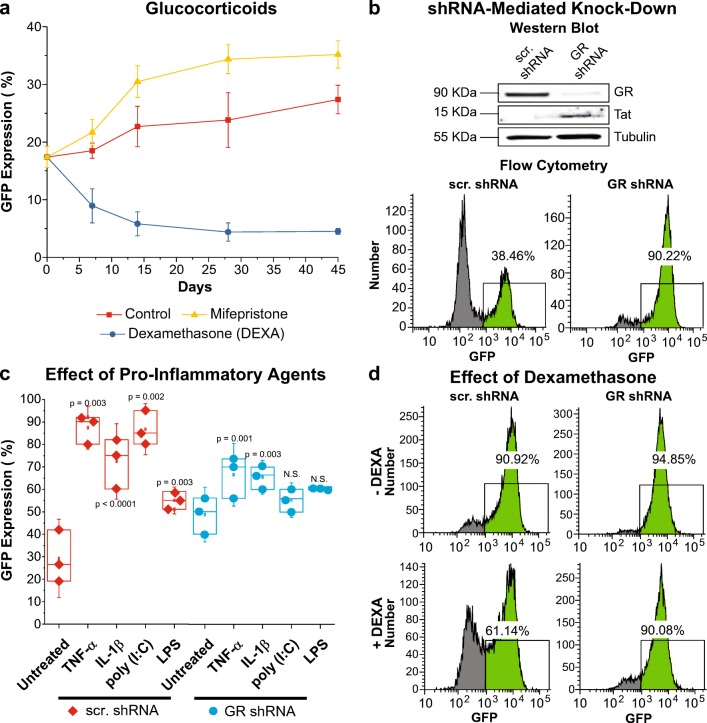
*Activation of GR induces HIV latency.****a****HC69 cells were cultured in the absence (control; red squares) or presence of* DEXA (blue circles; 1 μM) or mifepristone (yellow triangles; 60 nM) for 45 days (X-axis). HIV expression (GFP) was measured by flow cytometry (Y-axis) at the time points indicated. *Error bars* represent the standard deviation of the sample (Excel) of three different experiments. **b** shRNA-mediated knockdown of GR. HC69 cells were superinfected with viral particles bearing scrambled shRNA or shRNA against GR. Western blot analysis of GR (90 KDa) and Tat (15 KDa) expression, using tubulin (55 KDa) as loading control, in WCE prepared from blasticidin (2 μg/mL)-resistant cells. Flow cytometry profiles of HC69 cells exposed to scrambled or GR shRNA. GFP^+^ cell populations are indicated in *bright green*, and the proportion of GFP-expressing cells is indicated in *percentage.****c****Effect of pro-inflammatory agents on HC69 cells bearing scrambled shRNA (red diamonds) or GR shRNA (blue circles). Cells were unexposed or exposed to* TNF-α (50 pg/mL), IL-1β (100 pg/mL), poly (I:C) (100 pg/mL), or LPS (1 ng/mL) for 16 h (X-axis), and GFP expression (%) was measured by flow cytometry (Y-axis). The *p*-values of statistically significant pair-sample t-tests (at 0.05 confidence level, where the difference of the sample means is significantly different from the test difference of zero) of three experiments (*n* = 3) comparing the unexposed vs. the exposed cells are shown. N.S. stands for *n*on-*s*ignificant. ***d*** Effect of DEXA on shRNA-bearing cells. HC69 cells *bearing scrambled shRNA or shRNA against GR were sorted for GFP*^*+*^*cells, and then untreated or treated with DEXA (1 μM). HIV expression was determined by flow cytometry analysis.* GFP^+^ cell populations are indicated in *bright green*, and the proportion of GFP-expressing cells is indicated in *percentage*

As shown in Fig. 3a, only DEXA (blue; 1 μM) was able to significantly reduce HIV expression in a long-term (from ~17% to ~3%), compared to the control, which increased progressively (red line; from ~17% to ~25%). In contrast, mifepristone (yellow line; 60 nM), increased HIV expression basal level from ~17% to ~45% during the same period of time.

All the other compounds produced minimal or transient inhibition of HIV reactivation (supplement Fig. 4 (a)). (s) DHMEQ (yellow line; 1 μg/mL) was significantly less effective than DEXA and only inhibited HIV expression during the first week. Similarly, (d) DHMEQ (blue line; 1 μg/mL) and the IKKγ inhibitory peptide (green line; 100 μM) had a strong inhibitory effect during the first week (similar to that of DEXA and (s) DHMEQ), but it was also subsequently reserved.

TGF-β1 (yellow line; 1 ng/mL) was also less effective than DEXA (from ~17% to only ~12% in 1 month), and this effect was reversed afterwards, and AEA (blue line; 2 μM) had no clear effect since it decreased HIV expression basal level slight during the first weeks, and then increased it (supplement Fig. 4 (b)).

### shRNA-Mediated Knockdown of GR Increases Basal Level of HIV Expression

In order to confirm the role of GR in regulating HIV transcription in microglial cells, we performed shRNA experiments. As expected, knocking down the expression of GR in HC69 cells induced HIV reactivation (Fig. 3b). Cells treated with scrambled shRNA, expressed HIV up to ~38%, whereas cells treated with GR shRNA expressed HIV up to ~90%. Western blot analysis on WCE showed that GR shRNA knocked GR protein expression down significantly, compared to scrambled shRNA, as demonstrated by the marked reduction in the GR band intensity, and that HIV Tat protein expression increased in the cells bearing the GR shRNA, as demonstrated by the higher intensity of the Tat band (Fig. 3b).

In order to investigate the capacity of cells expressing low levels of GR to reactivate HIV by pro-inflammatory agents, cells bearing either scrambled shRNA or GR shRNA were exposed to TNF-α (50 pg/mL), IL-1β (100 pg/mL), poly (I:C) (100 pg/mL), or LPS (1 ng/mL) for 16 h (Fig. 3c). As expected, each of these agents was able to induce HIV expression significantly over basal levels in the control (scrambled shRNA-treated) cells.

In the GR shRNA cells, the responses to TNF-α, IL-1β and poly (I:C) were inhibited compared to the control cells, even though basal HIV expression in these cells was higher than in the control cells (Fig. 3c). For example, HIV expression increased from ~30% up to ~ 85% with TNF-α and up to ~70% with IL-1β, whereas in GR shRNA cells, HIV expression increased from ~50% up to only ~ 65% with either TNF-α or IL-1β. By contrast, there was no impact of GR shRNA on responses to LPS compared to the scrambled shRNA control cells. It is notable that LPS is a much weaker inducer of HIV expression than the other agents in this system.

To get a better picture of the activation scale and viability of cells bearing either scrambled shRNA or GR shRNA exposed to these pro-inflammatory agents, we titrated each on these compounds in the dose range from 5 pg/mL to 10 ng/mL, and measured HIV expression and cell survival (supplement Fig. 5). These results show that cells with restricted GR expression have higher spontaneous HIV reactivation levels, suggesting GR acts a repressor. However, these cells also become restricted to inflammation-mediated increase of HIV expression likely due to the activation of inhibitory pathways that prevent an excessive pro-inflammatory response.

To verify that the inhibition of HIV by DEXA was due directly to activation of GR, GFP^+^-sorted HC69 cells bearing either scrambled shRNA or GR shRNA were cultured in the absence or presence of DEXA. We used GFP^+^-sorted cells to start with a homogenous population of HIV-expressing cells. The results (Fig. 3d) demonstrated unequivocally that DEXA inhibition requires a functional GR, since in the cells bearing GR shRNA, HIV expression decreased minimally (from ~95% to ~90%). In contrast, in control cells bearing scrambled shRNA, DEXA decreased HIV expression from ~91% to ~61%.

### DEXA Inhibits Secretion of TNF-α and Other Cytokines

**Fig. 4:**
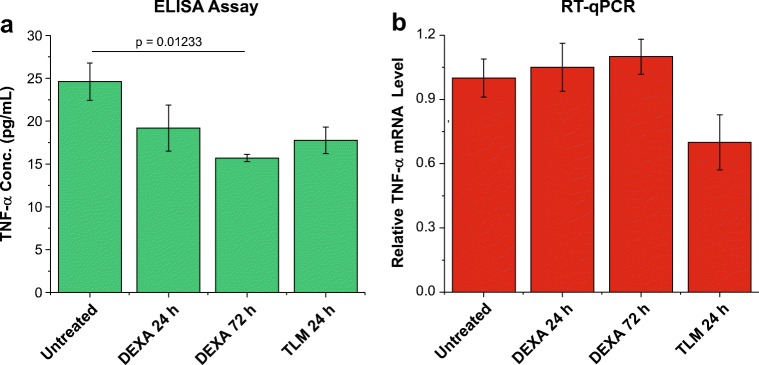
DEXA effect on TNF-α expression. HC69 cells were untreated or treated with DEXA (1 μM) for 24 or 72 h (X-axis) prior to collection of cell culture supernatant and isolation of total mRNA to measure **(a)** TNF-α secretion (green; Y-axis) by ELISA, and **b** relative TNF-α mRNA level (red; Y-axis) by RT-qPCR, respectively. TLM (25 μM) treatment for 24 h was used as positive control. The p-value of a statistically significant pair-sample t-test (at 0.05 confidence level, where the difference of the sample means is significantly different from the test difference of zero) of three experiments (n = 3) comparing the untreated vs. the DEXA 72 h-treated cells is shown

As described above, the population of HC69 cells that expresses HIV (GFP^+^) has higher levels of TNF-α mRNA and secretes higher quantity of TNF-α protein than the GFP^−^ population (Fig. 2). To evaluate whether DEXA is able to inhibit TNF-α expression in microglial cells, unsorted HC69 cells were exposed to DEXA (1 μM) for 24 or 72 h, and TNF-α protein and mRNA levels were measured. Treatment with DEXA decreased TNF-α secretion (green) from ~25 pg/mL down to ~18 pg/mL after 24 h and down to ~15 pg/mL by 72 h (Fig. 4a). The TNF-α expression inhibitor TLM (Deng et al. 2003), had a comparable to the effect (Fig. 4a). To our surprise, DEXA did not reduce TNF-α mRNA levels (red), whereas TLM did reduce TNF-α mRNA levels (Fig. 4b). This result suggests that the block to HIV expression exerted by DEXA is partially due to its inhibitory effect on TNF-α secretion.

To investigate whether DEXA is able to inhibit secretion of other cytokines, GFP^+^ and GFP^−^ sorted HC69 cells were unexposed or exposed to 1 μM of DEXA for 96 h prior to collecting the supernatant. The expression of 36 different cytokines in the culture media was measured using a human cytokine array kit (R&D Systems) (supplement Fig. 6). As expected, TNF-α (blue) was among the most highly inhibited, but the expression of other cytokines is also compromised in the presence of DEXA (red) in both GFP^+^ and GFP^−^ cells, compared to the expression in the absence of DEXA (green). Hence, the effect of DEXA on basal HIV expression, while largely driven by TNF-α, could also be due to its impact on multiple other cytokines.

### DEXA Induces GR Recruitment to the HIV Promoter and its Phosphorylation on S226

**Fig. 5:**
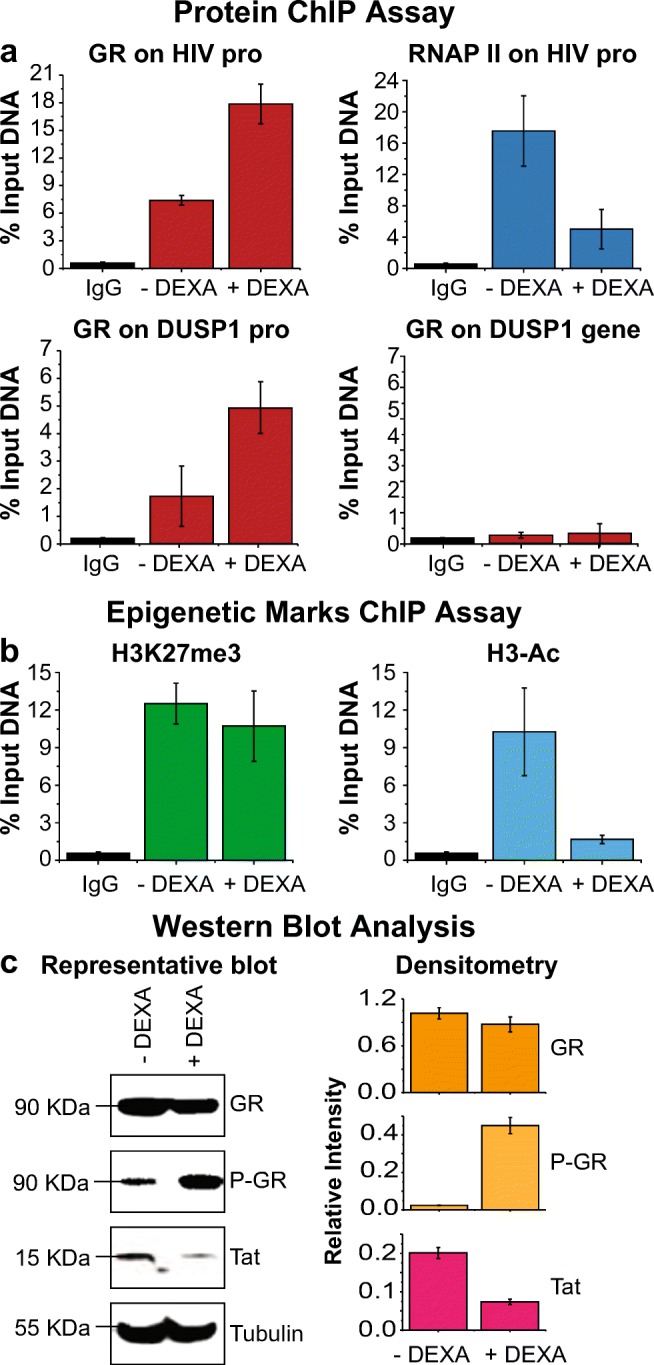
DEXA induces GR recruitment to the HIV promoter region. **(a** and **b)***ChIP analysis of the levels of GR, RNAP II, H3-Ac, and H3K27me3 in the HIV promoter region. GR recruitment to the DUSP1 gene promoter was quantified as positive control, and to an unrelated DUSP1 intragenic region as negative control. HC69 cells were untreated or treated with 1 μM DEXA (X-axis) for 16 h.* Equal numbers of unsorted HC69 cells undergoing spontaneous HIV expression were used for this experiment. *The abundance of GR and RNAP II****(a)****and of the epigenetic markers H3-Ac and H3K27me3****(b)****at the HIV promoter was calculated as % of input DNA (Y-axis). In each case, the e*rror bar represents the standard deviation of the sample (Excel) of three different experiments*.****c*** WCE Western blot analysis. A representative Western blot of GR (90 KDa), P-GR (90 KDa), and Tat (15 KDa) expression, using tubulin (55 KDa) as loading control, in WCE isolated from HC69 cells untreated or treated with DEXA (1 μM). Band intensity (densitometry) was determined by ImageJ (NIH). *Error bars* in the densitometry analysis (Y-axis), which was performed using blots from at least three similar Western blot experiments (X-axis), represents the standard deviation of the sample (Excel) of three different experiments

ChIP experiments were performed to establish whether there could also be a direct impact of GR on HIV transcription. Unsorted HC69 cells were untreated or treated with 1 μM DEXA for 16 h prior to performing ChIP analysis to measure recruitment of GR to the HIV promoter (−116 / +4 region, upstream of Nuc 1 region). As shown in Fig. 5a, GR (red) levels at the HIV promoter increased ~3 fold upon treatment with DEXA. This increase is comparable to that at the DUSP1 gene promoter, which was used as a positive control (Reddy et al. 2009; Shipp et al. 2010). There was no increase in GR at an unrelated internal region of the DUSP1 gene, demonstrating the specificity of the ChIP measurements.

Levels of RNAP II (dark blue) at the same site of the HIV promoter were inversely proportional to levels of GR, and decreased ~3 fold (Fig. 5a) after DEXA treatment. The recruitment of GR at the HIV LTR in the presence of DEXA also occurred in concomitantly to a strong reduction in the epigenetic marker of activation H3-Ac (light blue) (Fig. 5b). The abundance of the epigenetic marker of repression H3K27me3 (green) remained constant (Fig. 5b). Therefore, the ChIP data suggests that recruitment of GR directly blocks HIV transcription through the recruitment of a repressor complex that specifically de-acetylates histone 3.

Western blot analysis of WCE isolated from untreated and DEXA-treated cells revealed that DEXA-mediated recruitment of GR to the HIV promoter may be associated with phosphorylation of nuclear GR at S226 (P-GR), an activation signal which, along with phosphorylation of other serine residues, affects GR binding to and transcriptional regulation of different target genes (Blind and Garabedian 2008). After treatment of cells with DEXA, cellular P-GR (light orange) levels, but not GR (dark orange) levels, increased ~8 fold (Fig. 5c), suggesting that GR activation and tethering to the HIV promoter is associated to its phosphorylation. As a control, we showed that HIV Tat protein (pink) levels are reduced ~4 fold in the presence of DEXA (Fig. 5c), consistent with the repression of HIV transcription. Tubulin blotting was used as loading control.

### TNF-α Inhibitors Reduce HIV Gene Expression in Microglial Cells

To confirm that TNF-α released from microglial cells contributes to reactivation of latent HIV, we used two other TNF-α inhibitors, NaN and NaC, in addition to TLM (Fig. 4), alone or in combination at 12.5 μM each with DEXA at 1 μM for 15 days. NaN and NaC block TNF-α release (Bissonnette et al. 1995), while TLM, inhibits TNF-α production (Deng et al. 2003). In contrast, DEXA binding to GR results in receptor activation, leading to suppression of TNF-α induced gene expression (van der van der Velden 1998).

**Fig. 6:**
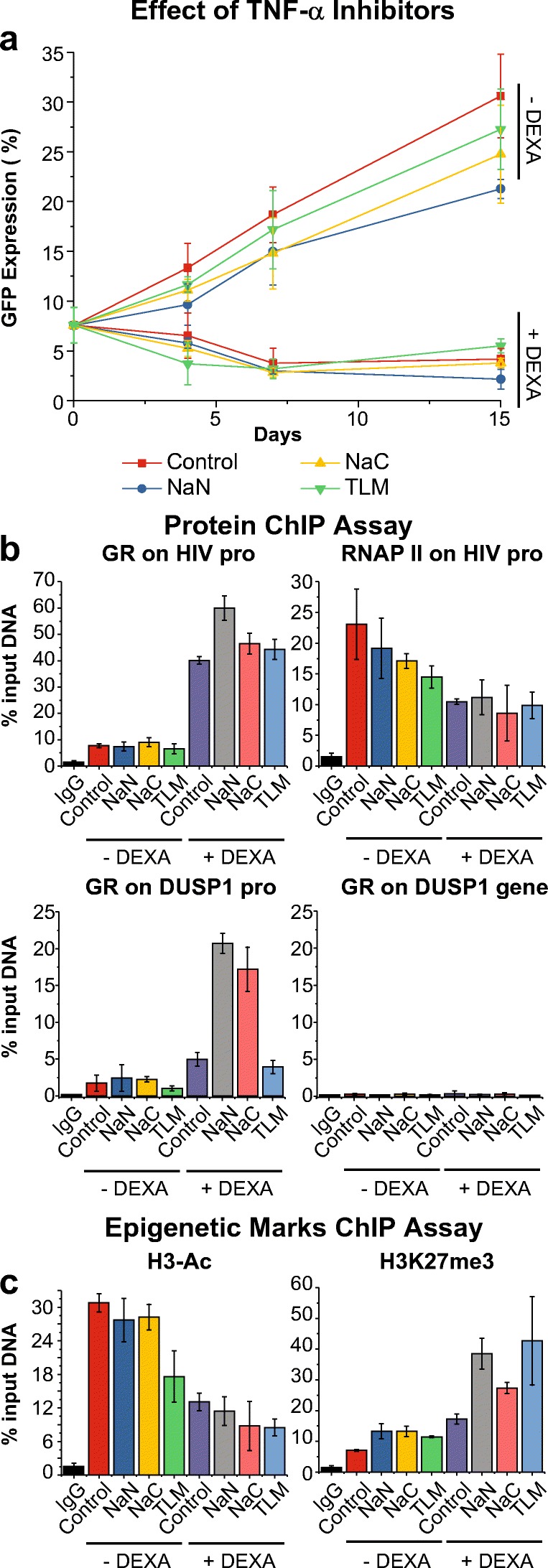
Effect of TNF-α inhibitors. **a** GFP^−^-sorted HC69 cells were untreated (Control; red squares) or treated with NaN (blue circles; 12.5 μM), NaC (yellow triangles; 12.5 μM), or TLM (green triangles; 12.5 μM) in either the absence or the presence of DEXA (2.5 μM) during two weeks (X-axis). GFP expression (Y-axis) was determined by flow cytometry at different time points. **(b** and **c)***ChIP analysis of the levels of GR, RNAP II, H3-Ac, and H3K27me3 in the HIV promoter region. GR recruitment to the DUSP1 gene promoter was quantified as positive control, and to an unrelated DUSP1 intragenic region as negative control. HC69 cells were untreated (Control; red) or treated with 12.5 μM NaN, 12.5 μM NaC, 12.5 μM TLM in either the absence or presence of 2.5 μM DEXA (X-axis) for 96 h.* Equal numbers of GFP^+^-sorted HC69 cells were used for this experiment. *The abundance of GR and RNAP II****(b)****and of the epigenetic markers H3-Ac and H3K27me3****(c)****at the HIV promoter was calculated as % of input DNA (Y-axis). In each case, the error bar* represents the standard deviation of the sample (Excel) of three different experiments

Each of these TNF-α inhibitors was able to slightly reduce spontaneous HIV expression in the absence of DEXA (Fig. 6a). For example, after 2 weeks, ~33% (red) of the untreated control cells were reactivated whereas ~27% of theTLM-treated cells (green), ~24% of the NaC-treated cells (yellow), and ~21% of the NaN-treated cells (blue) were reactivated. Similarly, each of these compounds enhanced DEXA blocks to HIV expression (Fig. 6a).

To confirm that inhibition of TNF-α expression in the presence of DEXA contributes to reducing HIV expression, we treated equal numbers of the GFP^+^-sorted HC69 cells with 12.5 μM NaN, 12.5 μM NaC or 12.5 μM TLM alone or in combination with 1 μM DEXA for 96 h, followed by ChIP analysis of GR, RNA pol II, H3-Ac, and H3K27me3. As shown in Fig. 6b, treatment with the TNF-α inhibitors (blue, yellow, and green) did not alter the abundance of GR at the HIV promoter, compared to the untreated control (red). By contrast DEXA (purple) induced a ~4-fold increase in GR abundance. The combination of DEXA and the TNF-α inhibitors somewhat increased GR occupancy of the HIV LTR (NaN ~6-fold, grey; NaC ~4.5-fold, light red; TLM ~4.5-fold, light blue). As in Fig. 5a, we measured GR occupancy at the DUSP1 gene promoter as positive control, and at an unrelated internal region of this gene as negative control.

The TNF-α inhibitors were able to slightly reduce the abundance of RNAP II at the HIV promoter (Fig. 6b). In combination with DEXA, which by itself reduced the abundance of RNAP II at the HIV promoter ~2.2 fold, no additional reduction of RNAP II at the HIV promoter was observed in the presence of the TNF-α inhibitors.

Changes in the epigenetic landscape mirrored the changes in GR levels at the HIV LTR. Only TLM was able to reduce the abundance of H3-Ac at the HIV LTR (from ~30% down to 18% (Fig. 6c). TLM in combination with DEXA (which alone reduced the abundance of H3-Ac ~2.3 fold), reduced H3-Ac levels by ~3.3 fold. The other two inhibitors, alone or in combination with DEXA, only had minimal effect.

The three inhibitors by themselves only had a modest effect on methylation of H3K27 (between 1.5 to 2-fold increase), similar to that of DEXA alone. However, combinations of DEXA and the TNF-α inhibitors resulted in pronounced increases in the level of H3K27me3 at the HIV promoter (~ 4 to 6 fold) (Fig. 6c), demonstrating enhanced epigenetic silencing under these conditions.

### Regulation of HIV Basal Level of Expression by TNF-α Inhibitors and DEXA Is Observed in Multiple Clonal Populations

**Fig. 7:**
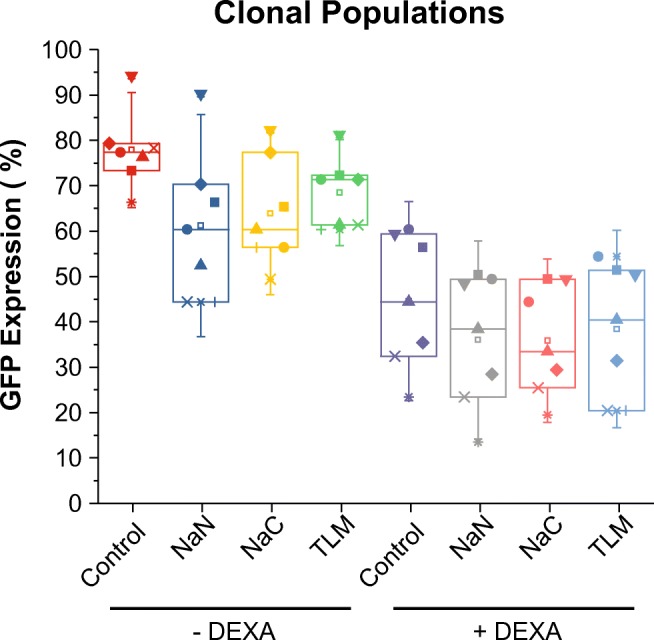
Activation of GR with or without inhibition of TNF-α induces HIV latency in multiple μglia/HIV clones. GFP^+^-sorted HC69 and the μglia/HIV clones (each represented by a different geometric shape) were untreated or treated (X-axis) with the TNF-α inhibitors NaN, NaC and TLM, each at 25 μM, in either the absence or presence of DEXA at 2.5 μM. GFP expression was measured by flow cytometry (Y-axis)

To demonstrate that spontaneous HIV reactivation was not unique to the HC69 clone, similar inhibition experiments were performed in 4 other clones isolated, as previously-described (Alvarez-Carbonell et al. 2017; Garcia-Mesa et al. 2017) (Fig. 7). Treatment of GFP^+^-sorted cells with the TNF-α inhibitors NaN, NaC, and TLM caused a 20 to 18% reduction in HIV expression for each of the microglial clones. DEXA reduced basal HIV expression by 35% on its own, and when paired with NaN, NaC or TLM, it caused an average reduction of between 38 and 45%, among all clones (Fig. 7).

We conclude that DEXA is a potent blocker of HIV reactivation with a dual mode of action consisting of a direct effect on blocking HIV transcription and an indirect effect on HIV expression by also blocking autocrine reactivation of cells mediated by TNF-α.

## Discussion

### Human Microglial Cell Models for HIV Latency

It is well known that HIV establishes transcriptionally silent infections in resting memory T cells, which allows the virus to persist in patients undergoing anti-retroviral therapy (ART) (Mbonye and Karn 2017). Much less is known about whether HIV establishes latency in myeloid cells and microglial cells in patients, and whether this has a clinical significance. Nonetheless, it is striking that, even in the era of ART, viral persistence in brain seems to be associated with low basal level of transcriptional expression, giving rise to HAND in 30 to 50% of infected individuals (Heaton et al. 2010, 2011, 2015; Chan and Brew 2014; Nightingale et al. 2014; Sacktor et al. 2016; Calcagno et al. 2017). Strong evidence supports the notion that microglia are the only resident cells in the brain parenchyma that can productively be infected with HIV, serving as a viral reservoir in the CNS (Chen et al. 2017).

We have developed models of HIV latency in immortalized human microglial cells (hμglia/HIV) (Garcia-Mesa et al. 2017). Using these models, we demonstrated previously that the TLR3 pathway is uniquely activated in microglia to promote HIV emergence from latency (Alvarez-Carbonell et al. 2017), and that enzymatic members of the CoREST repression complex are key regulators of HIV latency in microglia (Llewellyn et al. 2017).

One important feature of μglia/HIV cells, which has not been observed in similar models of T cells or monocytes, is their high rate of spontaneous HIV reactivation and hence increased basal level of HIV expression in culture. To minimize this effect, we investigated a variety of cell culture conditions that more closely recapitulate CNS physiological environment and result in long-term HIV silencing.

The CNS, unlike the periphery, requires a continuous supply of glucose because, due to the blood brain barrier, the brain evolved as an inefficient metabolizer of other energy suppliers such as fatty acids, amino acids or ketones (Siesjo 1978). In normal brain, glucose is metabolized almost completely and very efficiently to CO_2_, maintaining glucose concentrations in CNS between 4 to 6 mM, in CSF between 2 to 3 mM, and in the extracellular matrix at 1.5 mM (Ghosh et al. 2017). Therefore, it is expected that at glucose levels above these ranges, cells may experience a state of hypermetabolism. Our results (Fig. 1d) support this idea, since at high glucose concentration (25 mM), basal HIV expression is higher likely due to an unnecessary increase in microglia metabolic activity. Theoretically, glucose concentrations lower than 1 g/L (5.55 mM), as detected in the extracellular matrix, should promote microglial growth in culture and, therefore, lower HIV basal level of expression, however, glucose concentrations below 2.5 mM (~ 0.5 g/L), the glucose level in BrainPhys (Bardy et al. 2015), significantly induced cell death (supplement Fig. 1). The BrainPhys medium, which is more representative of the CNS extracellular environment because it increases the proportion of synaptically active neurons, is being used for growing primary neurons, differentiating and maturing human ES/iPS cell-derived neurons, microelectrode array-based recording of neuronal activity, live in vitro fluorescent imaging, and transdifferentiation of somatic cells to neurons (Bardy et al. 2015).

Cell-type specific gene expression data analysis shows that microglia express the full complement of genes required for glycolytic as well as oxidative energy metabolism (Zhang et al. 2014; Ghosh et al. 2017), confirming the capacity of microglia to generate ATP by both glycolytic and oxidative pathways. Thus, HEPES, which has been reported as a source of H_2_O_2_ production (Zigler Jr. et al. 1985; Bagger et al. 1987; Lepe-Zuniga et al. 1987; Marucco et al. 2015), induces HIV expression (supplement Fig. 2), most likely through activation of microglia oxidative pathways.

The content of FBS in the cell culture medium is another factor that can alter cell metabolic activity. FBS contains a myriad of growth factors, which are essential for the maintenance and growth of cultured cells as well as small molecules like amino acids, sugars, lipids, and hormones (Shah 1999; Even et al. 2006). Our results (supplement Fig. 3) suggest that a delicate balance of FBS contents in necessary to promote HIV silencing, while maintaining cell viability. Further studies may provide a superior formulation using only those components that can help achieve both goals at the same time, that is, long-term HIV latency and cell growth.

### Autocrine Induction of HIV Expression by TNF-α

Microglia infection by HIV is strongly associated with high production of cytokines, particularly TNF-α (Wilt et al. 1995; Verma et al. 2010), which has also been shown to be induced by Tat secreted by infected macrophages and astrocytic cells (Chen et al. 1997). Our results using an HIV-latently infected microglia model confirmed this observation, since cells actively expressing HIV, either spontaneously, or induced by the pro-inflammatory agent poly (I:C), expressed significantly higher amounts of both mRNA and protein TNF-α than cells with low basal level of HIV expression (Fig. 2).

Studies performed with HIV patients have demonstrated that, although there are rare microglial cells that appear to be infected, the majority of the pathophysiology is associated with numerous uninfected microglial cells that are activated and express TNF-α (Tyor et al. 1992). Furthermore, the abundance of TNF-α mRNA correlates with HAD severity (Wesselingh et al. 1993). Ex vivo studies have also demonstrated an association between TNF-α production and an increase in HIV replication. For example, when purified human microglial cells from adult brain were subjected to a neurotropic HIV infection in the presence of neutralizing TNF-α antibodies, there was delayed p24 expression and syncytium formation, and supernatants from these cells were less toxic to rat oligodendrocytes than supernatants from control cells (Wilt et al. 1995).

### Direct Repression of HIV Transcription by Glucocorticoids

Since manipulating cell culture conditions was unable to reduce spontaneous HIV reactivation in μglia/HIV cells in a long-term, we investigated a wide range of compounds that have been reported to inhibit macrophage activation. These reagents included the synthetic GC DEXA, and NF-κB and macrophage activation inhibitors. Each of these inhibitors had only a short-term effect on HIV expression, and their effect dissipated after a week.

DEXA, a GR agonist, was the only reagent capable to induce long-term HIV silencing. This is consistent with previous reports that DEXA can also repress the expression of pro-inflammatory genes including IL-1β and TNF-α (Newton 2000), and with our own data demonstrating that DEXA, by itself, was capable of inhibiting TNF-α secretion down to levels comparable to that of the TNF-α production inhibitor TLM.

In agreement with a direct role for GR in HIV regulation in microglia, we found that the steroid antagonist mifepristone slightly induced HIV expression. Mifepristone has been found to increase TNF-α production in myeloid cells (Lazar Jr. et al. 1992).

GC response element was identified many years ago in the HIV genome (Soudeyns et al. 1993), and inhibition of HIV expression by GCs in various cell lines, including macrophages, has been previously reported (Kino et al. 2000; Hanley and Viglianti 2011), however the physiological significance of GR on HIV expression and its role in mediating HIV expression in microglial cells have not been previously investigated. In an important extension of the earlier studies, we have used ChIP assays to demonstrate that GR is directly associated with the HIV LTR in suppressed microglial cells. Furthermore, GR abundance at the HIV promoter is significantly increased after treatment with DEXA. In multiple cell lines, GR is phosphorylated at S211 and S226 upon treatment with DEXA (Avenant et al. 2010; Lynch et al. 2010). Similarly, increased P-GR levels, rather than changes in GR levels, correlates with HIV reactivation. Together, these results suggest that GR is a direct repressor of HIV transcription.

We suggest that DEXA is a strong candidate therapeutic agent for limiting HIV induced neuropathy in HAND and HAD patients. DEXA has been used as a neuroprotective agent in other neuropathic conditions. For example, DEXA displayed anti-inflammatory effects against *Borrelia burgdorferi*-induced inflammation in glial and neuronal cells by significantly reducing inflammatory mediators in the CSF and inflammatory neurodegenerative lesions in the brain and spinal cord of treated Bb-infected animals (Ramesh et al. 2017). In Parkinson disease rat models, delivery of CD163-targeted liposomes containing DEXA provided protection against 6-OHDA-induced dopaminergic neurodegeneration, which correlated with a distinctive microglia response (Tentillier et al. 2016).

Theuse of TNF-α specific inhibitors together with DEXA has an even stronger inhibitory effect on HIV reactivation, and correlates with increased GR recruitment to the HIV promoter and epigenetic changes consistent with increased HIV suppression. This combination of agents may also be usefully explored as novel therapeutics for palliating HAND.

## Electronic Supplementary Material

ESM 1 *Effect of glucose on microglial cell viability. HC69 cells were cultured in the absence or presence of increasing concentrations of glucose (X-axis). Propidium iodide (PI) staining viability was measured and reported in % (Y-axis). E*rror bars represent the standard deviation of the sample (Excel) of three different experiments (EPS 994 kb)

High resolution image (PNG 32 kb)

ESM 2 HEPES induction of spontaneous HIV reactivation in microglia. Flow cytometry analysis of HIV expression in HC69 cells exposed or not to HEPES for either 7 or 14 days. GFP^+^ cell populations are indicated in *bright green*, and the proportion of GFP-expressing cells is indicated in *percentage (EPS 1408 kb)*

High resolution image (PNG 93 kb)

ESM 3 Effect of FBS on HIV basal level. HC69 cells were cultured in the absence or presence of 0.2, 0.5, 0.8, 1, 1.5, 2, or 5% FBS (X-axis). HIV expression (GFP) was measured by flow cytometry (green), and cell viability by PI staining (red) (Y-axis) after 3 days. *Error bars* represent the standard deviation of the sample (Excel) of three different experiments (EPS 410 kb)

High resolution image (PNG 42 kb)

ESM 4 *Effect of NF-κB and macrophage activation inhibitors.****(a****and****b)****HC69 cells were cultured in either the absence (control; red square) or the presence of the***(a)***NF-κB inhibitors* (d)-DHMEQ (blue circle; 1 μg/mL), (s)-DHMEQ (yellow triangle; 1 μg/mL) or IKKγ inhibitor peptide (green triangle; 100 mM), or of the **(b)** macrophage activation inhibitors anandamide (blue circle; 2 μM), or TGF-β1 (yellow triangle; 1 ng/mL) for 45 days (X-axis). HIV expression was measured by GFP flow cytometry (Y-axis). *E*rror bars represent the standard deviation of the sample (Excel) of three different experiments (EPS 1244 kb)

High resolution image (PNG 106 kb)

ESM 5 Effect of pro-inflammatory agents on HIV in GR shRNA-bearing cells. HC69 cells *bearing either scrambled shRNA (red) or shRNA against GR (blue) were untreated or treated with indicated doses (X-axis) of****(a)****TNF-α,****(b)****IL-1b,****(c)****poly (I:C), or****(d)****LPS. HIV expression by flow cytometry analysis (bars) and viability by PI staining (lines) were determined (Y-axis). E*rror bars represent the standard deviation of the sample (Excel) of three different experiments (EPS 1749 kb)

High resolution image (PNG 207 kb)

ESM 6 DEXA effect on the expression of different cytokines. GFP^−^**(a)** and GFP^+^**(b)** HC69 cells were untreated or treated with DEXA (1 μM) for 96 h prior to collection of cell culture supernatant to measure cytokine secretion using the Human Cytokine Array Kit, Panel A (R&D Systems, #ARY005), and following the manufacturer instructions. Spot intensity (densitometry) was determined by ImageJ (NIH). Detected cytokines (X-axis) and the relative intensity (Y-axis) based on the control (green) are indicated. TNF-α is shown in blue for reference. *Error bars* represent the standard deviation of the sample (Excel) of three different experiments (EPS 1183 kb)

High resolution image (PNG 149 kb)
