# Supplementary figures and images for: The Glucocorticoid Receptor Is a Critical Regulator of HIV Latency in Human Microglial Cells
Source: J Neuroimmune Pharmacol. 2018 Jul 10;14(1):94–109. doi: 10.1007/s11481-018-9798-1 (PMC6394485; doi:10.1007/s11481-018-9798-1)

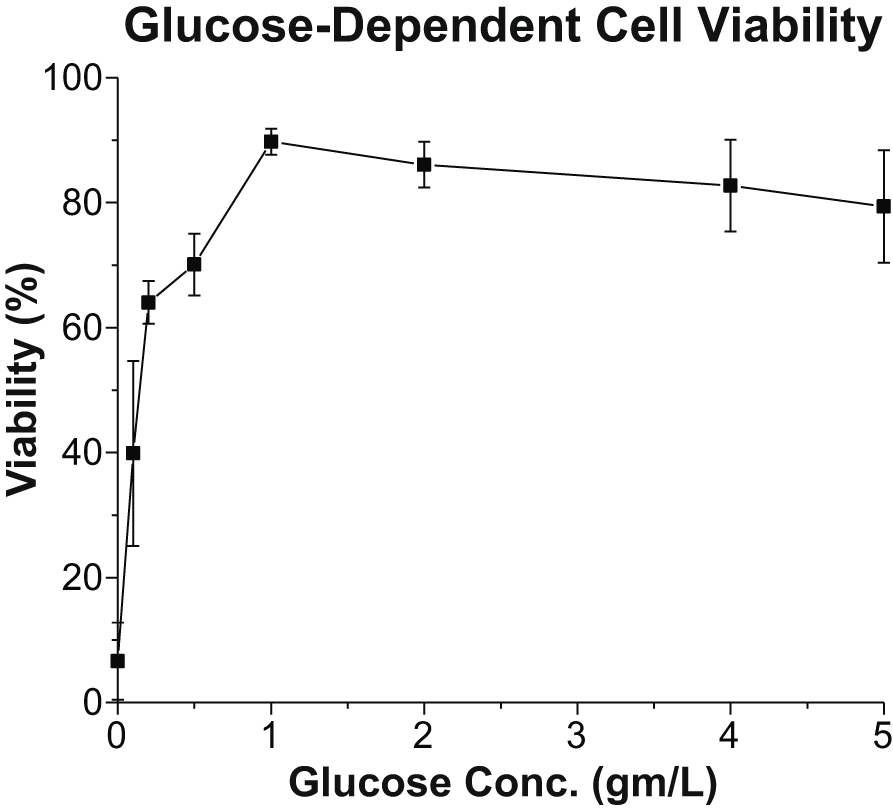

Supplement: Supplementary file 2 — High resolution image (PNG 32 kb) [file 11481_2018_9798_Fig8_ESM.png]

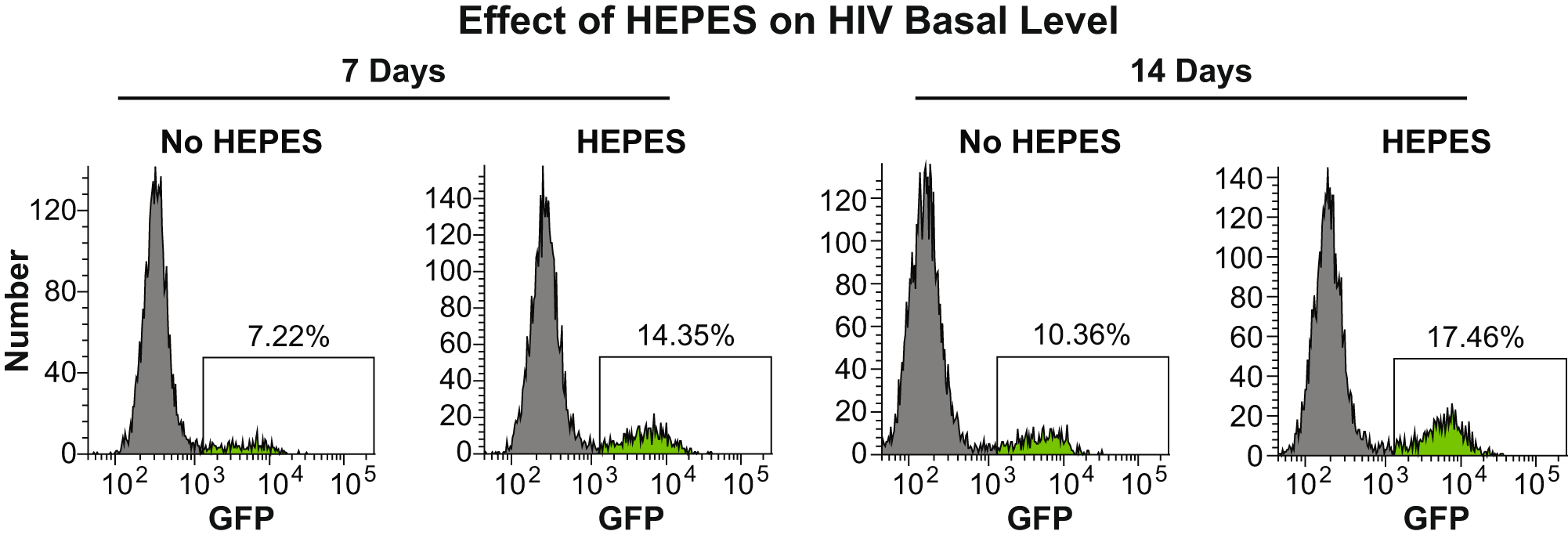

Supplement: Supplementary file 4 — High resolution image (PNG 93 kb) [file 11481_2018_9798_Fig9_ESM.png]

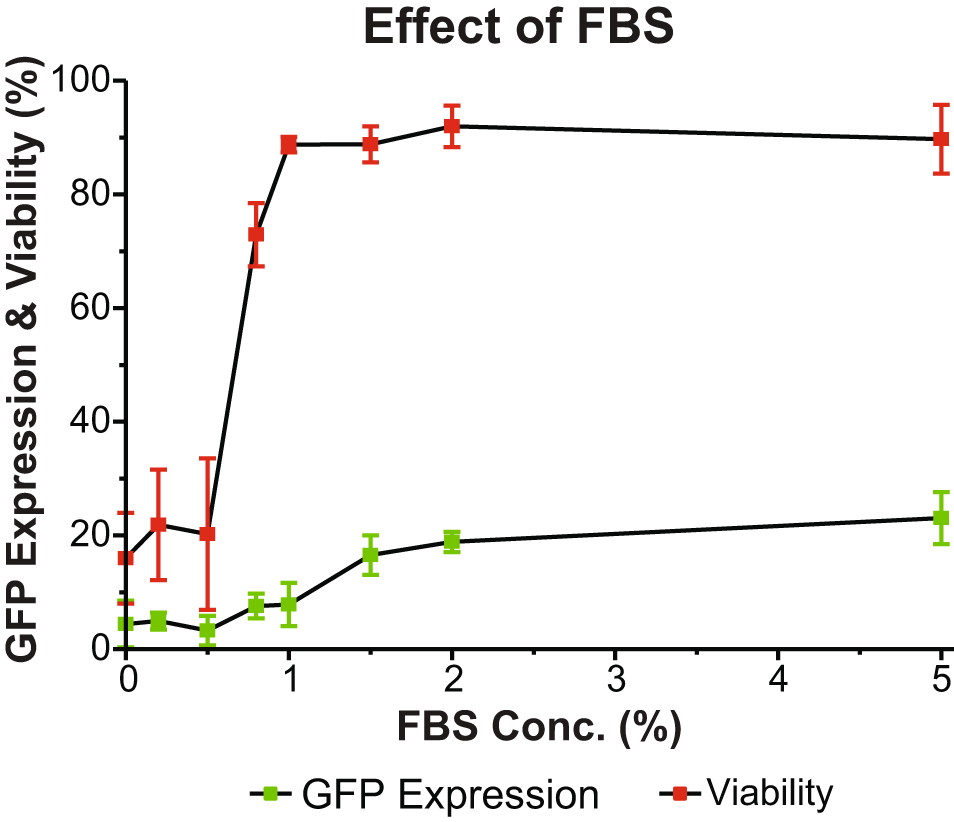

Supplement: Supplementary file 6 — High resolution image (PNG 42 kb) [file 11481_2018_9798_Fig10_ESM.png]

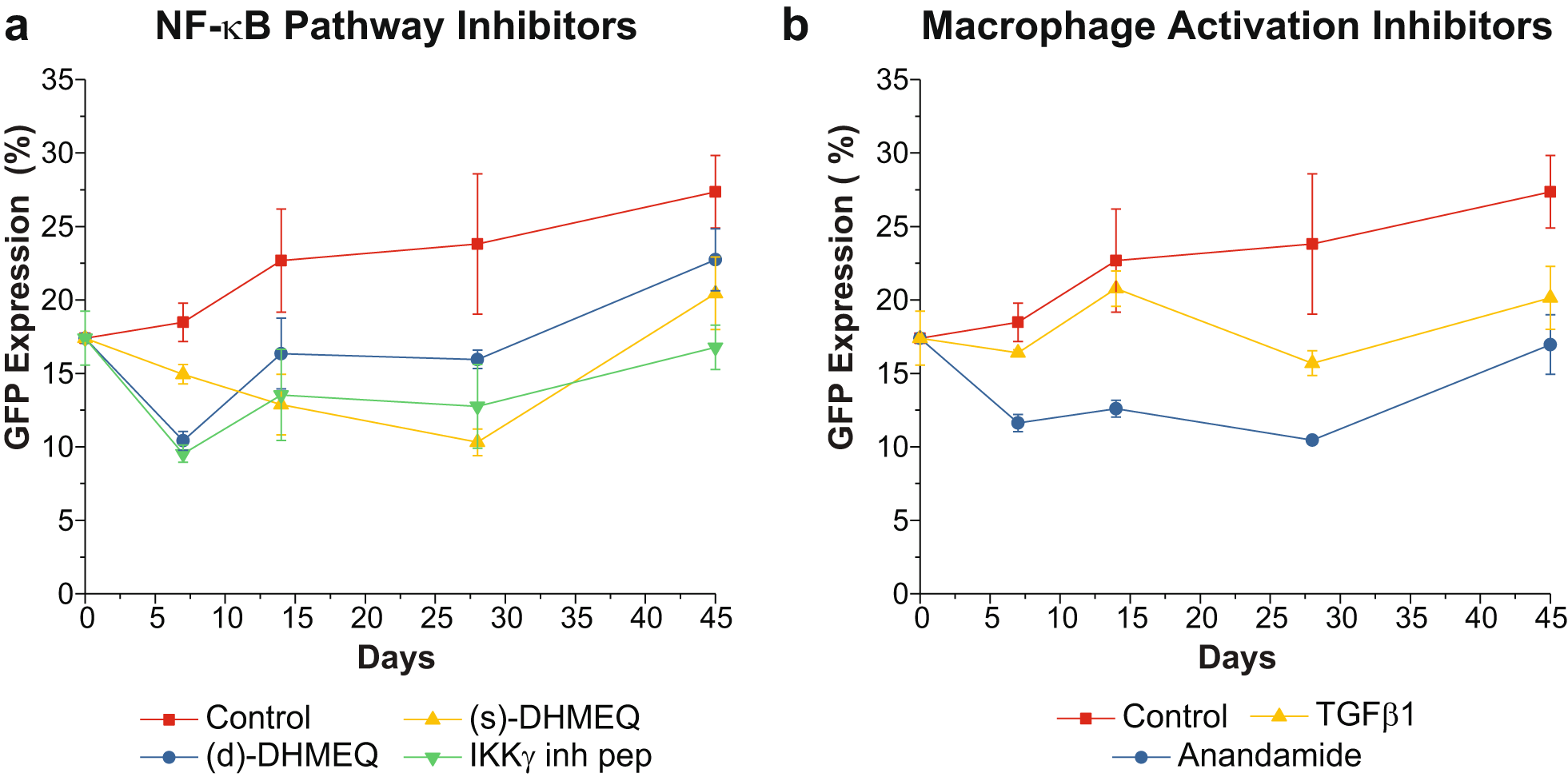

Supplement: Supplementary file 8 — High resolution image (PNG 106 kb) [file 11481_2018_9798_Fig11_ESM.png]

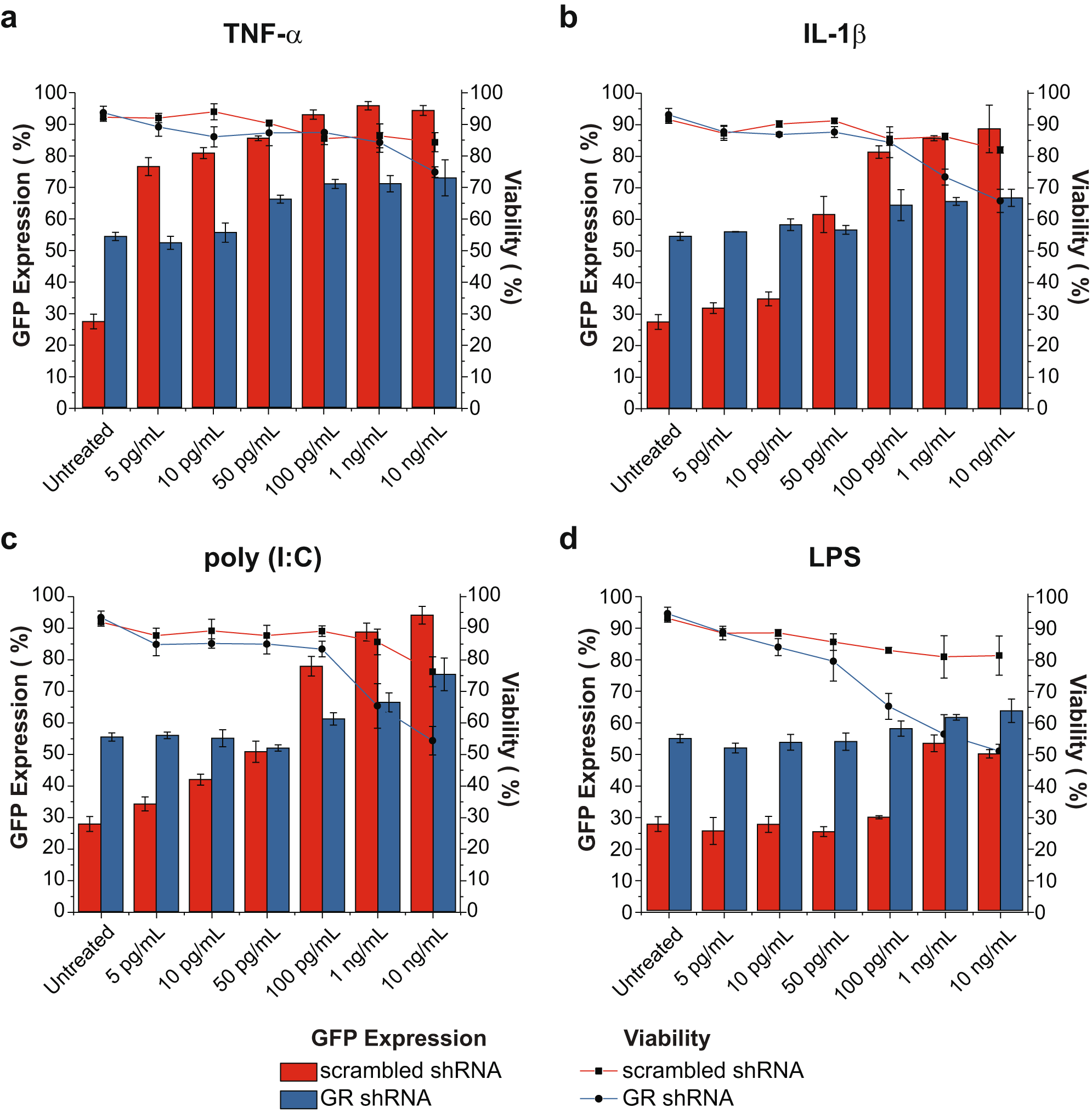

Supplement: Supplementary file 10 — High resolution image (PNG 207 kb) [file 11481_2018_9798_Fig12_ESM.png]

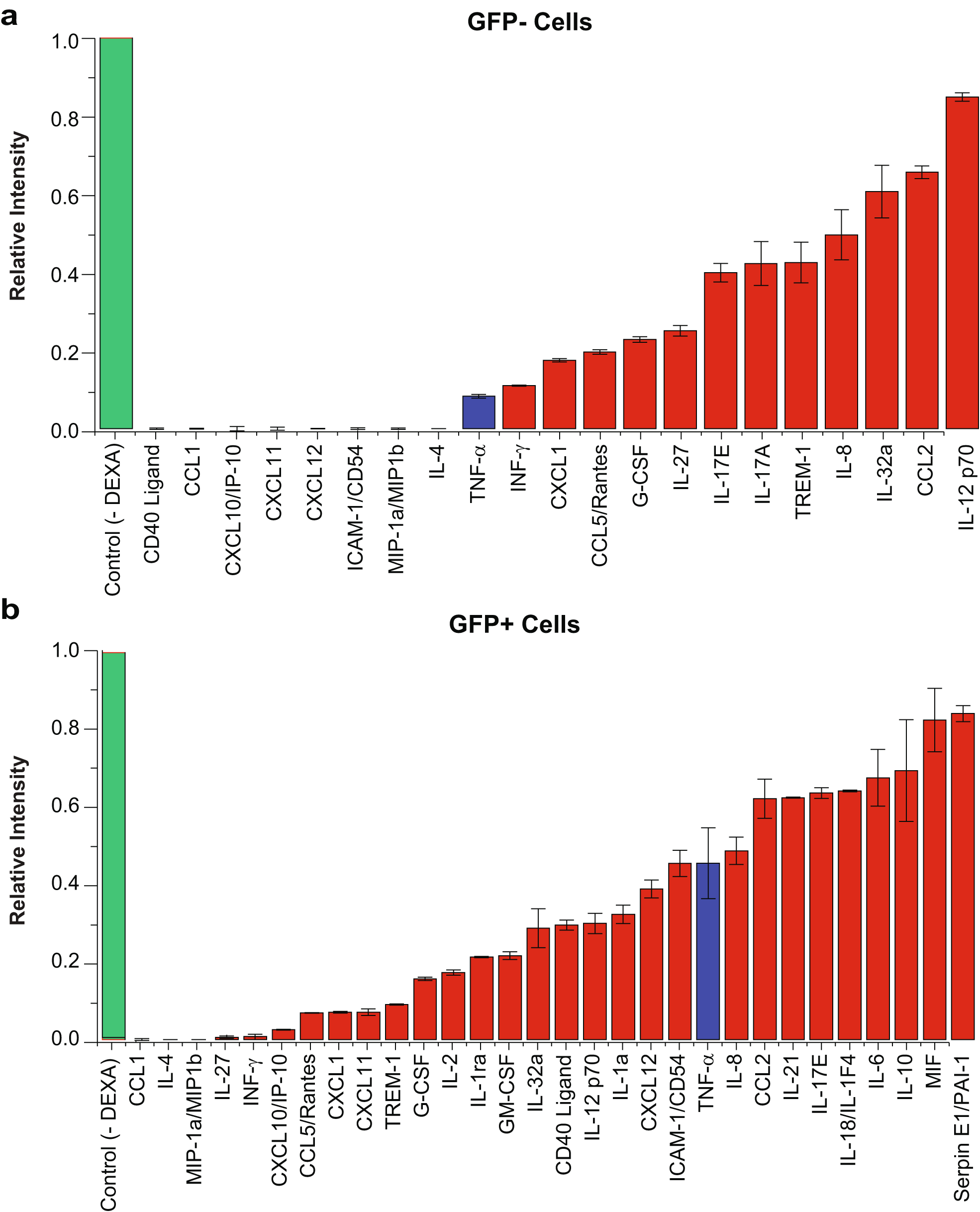

Supplement: Supplementary file 12 — High resolution image (PNG 149 kb) [file 11481_2018_9798_Fig13_ESM.png]
